# Responding to crisis: the intersection of police officer attitudes, organizational climate, and mental health crises

**DOI:** 10.1186/s12889-025-23832-8

**Published:** 2025-10-07

**Authors:** Therese L. Todd, Preeti Chauhan, Leah G. Pope, Amy Watson

**Affiliations:** 1 New York University School of Law, 110 W 3rd Street, New York, NY 10012 USA; 2 Psychology Department, John Jay College of Criminal Justice, 524 W 59th Street, New York, NY 10019 USA; 3 Department of Psychiatry, Columbia University Vagelos College of Physicians and Surgeons, New York, NY 10032 USA; 4 New York State Psychiatric Institute, 1051 Riverside Drive, New York, NY 10032 USA; 5 The School of Social Work, Wayne State University, 5447 Woodward Avenue, Detroit, MI 48202 USA

**Keywords:** Police officers, Implicit and explicit bias toward mental illness, Mental illness stigma, Police organizational climate, Mental health crisis response, Police officer decision-making

## Abstract

**Background:**

Police responses to people with serious mental illness (SMI) disproportionately result in negative outcomes, underscoring the importance of understanding the factors that may influence how police respond to mental health crises. Little research has examined officer attitudes toward mental illness, and no study has examined implicit bias toward mental illness among police officers or how bias and police departmental climate may impact outcomes in mental health crises. The present study assesses the role of explicit and implicit bias toward mental illness, as well as departmental climate, on police officer decision-making in a mental health crisis.

**Methods:**

Police officers in the United States (*n *= 198) were recruited from personal contacts, emails to leadership and administration in police departments, and Police1 (a policing blog). Police officers completed self-report questionnaires, two implicit association tests, a police organizational climate measure, and questions related to use of force, arrest, mental health transport, and no action decisions based on a written vignette of an officer encounter with a person with SMI.

**Results:**

Results demonstrate police officers who desired greater social distance from people with SMI were less likely to transport them to mental health services, whereas those who endorsed higher levels of microaggressions against people with SMI were more likely to transport them to services. Additionally, officers with more negative attitudes toward mental illness were less likely to take action in a mental health crisis. Finally, officer-related procedurally just organizational climate moderated the relationship between bias and officer decision-making: those with the lowest ratings of officer-related organizational climate, and highest levels of implicit bias toward people with SMI, were least likely to transport a person with SMI to services or take action in a mental health crisis.

**Discussion:**

Overall, this study has implications for how police respond to people with SMI in mental health crises and its findings may inform efforts to reduce adverse outcomes related to these interactions.

**Supplementary Information:**

The online version contains supplementary material available at 10.1186/s12889-025-23832-8.

## Introduction

Both individual and societal level factors contribute to the high levels of interactions between the police and people with serious mental illness (SMI), including a lack of access to mental health care, public intolerance for aberrant behavior, and officer attitudes and training [1]. These factors likely also influence how individuals are treated by the police and how these incidents are resolved. Consider a young man living with his parents who has become agitated. He is incoherent, delusional, and is convinced that his parents are not his parents. Every time they try to approach him, he becomes aggressive and tells them to get away from him. Out of concern and desperation, his parents call 9-1-1 to request help for their son. Officers are dispatched to the home. The resolution can range from a visit to the home with no action, to the son being arrested or transported to a hospital involuntarily, or at worst, officer use of deadly force.

Police officers routinely respond to mental health crisis calls such as this one and are asked to de-escalate situations involving psychological distress [2]. Despite departmental efforts to provide mental health crisis response trainings to officers, typically at most one 40-hour training with annual refreshers, (see [3] for an example training description) they receive relatively little mental health training compared to mental health professionals. Given that the United States lacks the adequate healthcare infrastructure to support and provide services for people with SMI, police officers will continue to play the role of mental health professional proxies. This is why it is critical to understand how police officers respond in crisis situations, and what may influence their responses.

Although research has examined public attitudes toward people with SMI [4–6], public attitudes may not generalize to police officers. Factors such as job training and duty-related interactions with people with SMI may result in different attitudes toward mental illness among police officers, which may impact their interactions with people with SMI and how these interactions are resolved. By understanding how these attitudes contribute to policing outcomes and behavior, the field will be better equipped to determine more optimal ways for how officers can respond to mental health crisis calls.

Organizational police climate [7], a systemic factor, may also influence the effect of biases on how police officers respond to mental health crises. For example, research shows that positive perceptions of department climate are associated with more positive officer behaviors and a service-oriented style of policing [8, 9]. However, it is unknown how police department climate impacts outcomes specifically for police interactions with people with SMI. The present study fills this gap by examining the role of individual officer attitudes and organizational climate on police officer decision-making in a mental health crisis incident presented through a written vignette.

We draw upon McLeroy and colleagues’ ecological model for health promotion, which shows how health behaviors and outcomes arise from multiple levels of the socio-ecological system, including intrapersonal, interpersonal, organizational, community, and policy factors [10]. In the case of police responses to mental health crises, police officers’ own attitudes, both those they report and the biases they carry unconsciously, set the stage for how they interpret and react to a situation. Those individual factors then exist within a broader culture, including police departmental climate, where formal policies, training priorities, leadership, and peers interact with each officers’ personal beliefs, attitudes, and behaviors. Finally, at the community-level, officers routinely respond to a variety of calls, including mental health crises. These crises may occur in a crowded public area, a private home, or in a region with limited mental health resources, all which impact what options are available and feasible for officers, affecting the trajectory of everyone involved. By understanding policing at these multiple levels of a public-health framework, it becomes clear that officers’ actions are multiply determined and can support, or undermine, outcomes in a crisis call. Taking this approach may lead to a better understanding of what interventions may be most useful for enacting change in crisis response at a population level.

## Outcomes of police interactions with persons with mental illness

It is estimated that approximately 10% of police encounters involve a person with SMI [2, 11, 12]. Responding to mental health crisis calls reportedly comprises a growing percentage of officers’ duties. For example, the Seattle Police Department reported that from the first half of 2017 to the first half of 2018, the department experienced a 27% increase in mental health crisis incidents [13]. These encounters can result in an array of suboptimal outcomes, including arrest and use of force.

Research examining the likelihood of arrest for people with SMI has yielded disparate findings. In 2019 in the U.S., just 3% of *all* police interactions resulted in arrest [14]. However, people with SMI are more likely to come into contact with the police than the general public through complaints, street checks, and tickets, and thus may be more likely to be arrested [15, 16]. A systematic review of 85 studies found that a much higher percentage of people with SMI (25%) are arrested and in the U.S., 29% received access to mental health services by way of police contact [17]. Using administrative data from Massachusetts, Fisher and colleagues [18] found that over a 9.5-year period those with a mental illness who used public mental health services were 1.62 to 5.96 times more likely to be arrested than those matched on age and gender in the general public across eight major offense categories. It also appears that people with SMI are more likely to be arrested for lower level offenses than those without mental illness [19]. Differences in rates may be due to methodology and analytic strategy, or department policies and location, still it is clear that people with SMI experience high levels of contact with the police.

Teplin [1] suggests that when people with SMI are arrested, they are not detained as a result of their offense, but due to extralegal factors related to their mental illness, such as community intolerance of the individual’s behavior, officers’ belief that the person would continue to cause problems, and having been turned away from hospitals. Officers’ discretion to arrest people with SMI may vary based on community expectations and the availability of mental health services or hospital resources within the community. Importantly, research has found police contact triggers a cycle of continued involvement in the legal system, including arrests and detention, for people with SMI [20, 21].

The most dangerous outcome in police interactions with people with SMI is use of force, which can be fatal. Use of force is estimated to occur in 1–2% of police encounters overall and 15–20% of police arrests [22, 23]. Research has demonstrated that people with SMI experience higher rates of use of force than the general public [24]. Further, mental illness is a significant predictor in police use of weapon-based force according to 4,267 use of force incidents between 1995 and 2008 in Victoria, Australia [25]. This study found that although people with SMI were more likely to experience use of force, those perceived to *not* have a mental illness were more likely to display abusive/violent language than those with SMI. This suggests that officers’ perception of mental illness may be a more powerful predictor of use of force than actual behavior and police officers found people with SMI more threatening, even in the absence of aggressive behavior. Saleh et al. [26] analyzed all deaths by the police in the U.S. (*n* = 1,099) in 2015. Findings revealed 23% of those killed by the police displayed signs of mental illness, and people with SMI were over seven times more likely to be killed by the police than those without a mental illness. The study also found that the presence of mental illness puts individuals at an increased risk of death across all races. However, Black individuals with mental illness had the highest death rate (i.e., 26 deaths per million), even when posing as less threatening. Thomas et al. [27], using a similar dataset found that among men killed by the police, Black men with mental illness were *significantly less likely to be armed* than White men with mental illness.

### Bias and behavior

Generally, people with SMI are negatively impacted by behaviors that stem from bias, including discrimination in everyday life (e.g., social isolation, poor interpersonal interactions, and negative media images) and structural discrimination (e.g., employment obstacles and discriminatory legislation) [28, 29]. Explicit bias is when individuals *are consciously aware* of and may openly disclose negative or harmful attitudes toward a specific group. However, individuals may also hold implicit bias, which is automatic in nature, where people *may not be consciously aware* of their negative attitudes toward a specific group. This may in turn, impact their behaviors or decision-making toward this group [30, 31]. Research across domains has shown a relationship between implicit biases (i.e., based on race, religion, sexual orientation, or body weight) and everyday judgments and behaviors such as reduced friendliness and increased social distancing [32]. In the context of policing, understanding the role of implicit biases on decision-making among officers is important given they are sometimes forced to make quick, automatic decisions in the line of duty [33]. Implicit Association Tests (IATs) are widely used to measure implicit bias, however there are mixed findings on IATs’ ability to predict behavior [34, 35]. Yet, evidence suggests, especially in well-designed studies, IATs can offer valuable insights into how bias contributes to discrimination [36, 37].

Some research has examined the relationship between bias toward people with SMI and behavioral outcomes and supports the notion that negative biases are related to undesirable outcomes for people with SMI. In a seminal 1973 study, job applicants who disclosed a history of psychiatric hospitalization were treated less favorably and told they were less likely to get the position in job interviews, providing early evidence of behavioral discrimination based on mental illness [38]. More recent research has found similar trends. Among medical residents, the presence of a psychiatric label resulted in a greater desire for social distance compared to a description of a person without a psychiatric diagnosis [39]. In a study with mental health professionals, other health professionals, undergraduates, and the general public, Peris et al. [40] assessed implicit and explicit biases of badness toward people with SMI. Results showed that although mental health professionals demonstrated relatively more implicit positive attitudes compared the other groups, implicit biases still predicted over-diagnosing alcohol dependence, major depression, and schizophrenia, whereas explicit bias did not. In another study, staff from Assertive Community Treatment (ACT) teams were assessed for implicit and explicit biases related to badness, blameworthiness, and helplessness of people with SMI [41]. ACT staff demonstrated an implicit preference for mental illness compared to physical illness on domains of goodness, competence, and innocence (*d* = −0.19 to −0.47). Yet, in response to a vignette, implicit biases significantly predicted higher endorsement of restrictive and controlling clinical interventions, such as invasive medication monitoring, money management, and hospitalization [41]. These results demonstrate even professional training and frequent contact with people with SMI does not appear to override implicit biases and their impact on diagnoses of behavioral health disorders. Importantly, clinical interventions, such as involuntary hospitalization, have substantial consequences on people’s liberties and autonomy. More research is needed to better to understand the implications of bias toward mental illness on decision-making and behavior, particularly in the context of the criminal legal system. The potential for biases to influence behavior highlights how officers’ attitudes may have real consequences for people in crisis. Within McLeroy’s ecological model [10], these individual-level factors form the first level of influence on mental health crisis response, framing how departmental factors may interact with these attitudes and shape officer decision-making.

### Police organizational climate

Police interactions with persons in crisis do not take place in a vacuum and exploring systemic factors that contribute to inequities may be more successful in bringing systemic change and reducing disparities than focusing on individuals [42, 43]. Addressing policies, protocols, training, and organizational climate may be more impactful in changing behaviors and outcomes for police interactions with people with SMI than focusing on officer-level factors. Plaut [43] explains that a sociocultural framework allows for the exploration of individual psychological experiences and behavior *and* the cultural context, including values and ideas, within which individual experiences occur. Together, these components lead to interpersonal experiences, power structures, institutional norms, and policies, all of which may be relevant to how police interact with people with SMI.

Police organizational climate is multi-dimensional and includes a wide range of cognitive and emotional components embedded in officers through departmental customs, traditions, and organizational policies and practices [7]. Tyler et al. [9] argues that the procedurally just nature of a police organization, such as beliefs about whether organizational leaders are legitimate and organizational rules and policies are morally sound, has implications for officer behavior. Organizational characteristics related to procedural justice have been predictive of rule adherence. Tyler et al. [9] suggests that when employees perceive their organizational environment as fair, employees will develop a strong identification with the organization, which will lead to engagement in desirable behavior and attitudes at and about work. The importance of procedural fairness in the workplace extends to various employment settings, including law enforcement [44]. In a study of both police officers and members of the military, Tyler et al. [9] found that perceived fairness of the organization and authority figures is predictive of deference to rules, specifically compliance, in-role performance, and voluntary deference to policies.

Other research has found that when officers feel they are treated in a fair manner, or work in a procedurally just climate, they are more likely to adopt a service-oriented style of policing [45], do extra work, and assist fellow officers [46]. A study of 786 police officers from a large, urban police department found that a procedurally fair organizational climate was positively related to organizational legitimacy and negatively related to officer cynicism and distress [47]. Further, organizational fairness was positively related to fulfilling job duties, doing extra work for their job, and endorsing a community model of policing, such as procedural justice tactics and rejecting excessive use of force.

Perceptions of departmental fairness and procedural justice have been associated with positive outcomes related to police conduct, such as a lack of adherence to a code of silence, disavowing corruption as justified, and less engagement in multiple forms of police misconduct [48]. Officers with higher perceptions of organizational procedural justice made more just managerial decisions, were more polite toward subordinates, had less citizen complaints, internal affairs investigations, and disciplinary charges [48]. Therefore, those who endorse higher levels of organizational procedural justice may be more likely to adhere to community policing practices in their interactions with community members. People with SMI are a group at the greatest risk for officer use of force and continued criminal legal involvement. Given that a procedurally just organizational climate has implications for officer behavior, it is important to consider how this may contribute to policing outcomes for people with SMI.

The present study builds on prior research by using a measure of organizational procedural justice to assess how departmental climate relates to officer decision-making involving people with SMI, and whether departmental climate moderates the relationship between individual-level attitudes and policing outcomes. Here, we suggest organizational climate, comprised of feelings of fairness of policies, supervisory decisions, and treatment compared to peers, operates as the organizational level context of McLeroy’s ecological model [10], moderating the impact of officers’ individual attitudes on their crisis responses.

### Present study

To our knowledge, no study has examined 1) the link between officers’ explicit and implicit attitudes toward mental illness and their decision-making in crisis contexts; 2) the association between police organizational climate and outcomes for people with SMI; or 3) the interaction between individual bias and departmental climate in mental health crisis encounters. Understanding police officers’ implicit attitudes toward people with SMI is particularly important given that research has demonstrated implicit attitudes may be more predictive of behavior than explicit attitudes [49]. Further, the climate of a department may moderate the relationship between implicit bias and policing outcomes in mental health crises. Although prior work has examined officers’ attitudes, departmental climate, and mental health crises separately, no research has integrated these levels of ecology to understand how they interact in police responses to mental health crises. As a result, there is a lack of a system-level perspective of how individual biases and organizational context operate to impact police decision‐making in a community-level context. Using a vignette design, the present study addressed the following research questions and corresponding hypotheses:

1. How does implicit bias relate to police officer decisions in encounters with people with SMI, particularly related to use of force, arrest, hospitalization, and no formal action?
  - Hypothesis: Implicit bias, over and above explicit bias, toward people with SMI will be a significant predictor of more restrictive and less proactive policing behaviors.
2. How does a procedurally just organizational police climate moderate the relationship between implicit bias and outcomes of a police encounter including severity of use of force, arrest, hospitalization, and no formal action decisions?
  - Hypothesis: A procedurally just organizational police climate will moderate the relationship between implicit bias and officer outcome decisions in a mental health crisis. Lower perceptions of a procedurally just organizational police climate and higher implicit bias will result in the endorsement of more restrictive and less proactive policing behaviors.

## Method

### Participants

Participants were active-duty police officers in the United States. We recruited police officers through personal contacts and direct emailing to police leadership and administration at 218 police departments (*n* = 185), and Police1 (*n* = 13), a U.S.-based policing blog. Police officers were paid $20 via an Amazon e-gift card for their time. Although we aimed to recruit a larger sample, challenges in recruiting active-duty police officers and resource limitations constrained our final sample size to 198. Despite this, a post hoc power analysis conducted in G*Power indicated that our regression models, which included 29 predictors, had 84% power to detect a medium effect size (f² = 0.15) at an alpha level of 0.05, suggesting sufficient power for our primary analyses [50]. However, smaller effects may go undetected increasing the risk of Type II errors.

Table 1 reports the demographic characteristics of the sample. The average age of participants was 41.8 years. The sample was majority male (75.8%)^1^ and White (88.9%). Due to small sample size, race/ethnicity variable was dichotomized into White and Other for multivariate analyses. The most common highest level of education among the sample was a bachelor’s degree (56.6%) and most officers made $100,000 or more, (78.8%). We collapsed income levels into three categories: less than $75,000, $75,000 - $99,999, and $100,000 or more. Finally, most officers lived in suburban areas (57.1%).

**Table 1:** Police Officer Characteristics

	Police Officers (*n* = 198)	
Demographic	*n*	Percent
Gender		
Male	150	75.8
Female	46	23.2
Non-binary	0	0.0
Other	2	1.0
Hispanic or Latino/a/x		
Yes	2	1.0
No	196	99.0
Race		
White	176	88.9
Black or African American	3	1.5
Asian/Pacific Islander	7	3.5
Native American	2	1.0
Biracial	4	2.0
Other	6	3.0
Highest education level		
Less than high school	0	0.0
High school or GED	4	2.0
Some college/Associates degree	36	18.2
Technical/Vocational/Professional schooling	2	1.0
Bachelor’s degree	112	56.6
Post-graduate degree	44	22.2
Yearly household income		
Less than $25,000	0	0.0
$25,000 - $49,000	3	1.5
$50,000 - $74,999	16	8.1
$75,000 - $99,999	22	11.1
More than $100,000	156	78.8
Department Geographic Region		
Urban	39	19.7
Suburban	113	57.1
Rural	46	23.2
	***M (SD)***	**Range**
Age (Years)	41.8 (9.7)	22–71

Table 2 shows the department characteristics within which the officers work. Most officers worked in an urban police department (49.5%) and department size of less than 100 officers (41.9%). For multivariate analyses, department size variable was categorized into the following categories: less than 100 officers, 101–500, 501–1,000, and more than 1,000 officers. Most officers had received Crisis Intervention Team (CIT) or another type of mental health training (75.3%), with the most common amounts of training being one week (45.6%) and annual (48.4%). Most officers worked in departments with designated mental health or CIT units/teams (64.1%), but only 11 officers were a member of these units. Lastly, the average number of Emotionally Disturbed Persons (EDPs) or mental health crisis calls officers responded to each week was somewhat evenly distributed.

**Table 2:** Police Officer Department-related Characteristics

Demographic	*n*	Percent
Police Department Geographic Region		
Urban	98	49.5
Suburban	70	35.4
Rural	30	15.2
Police Recruitment Source		
Email Distribution	185	93.4
Police1	13	6.6
Police Department Size		
Less than 100 officers	83	41.9
101–500 officers	37	18.7
501–1,000 officers	48	24.2
1,001–1,500 officers	24	12.1
1,501–2,000 officers	3	1.5
More than 2,000 officers	3	1.5
Crisis Intervention Team (CIT) or MH Training		
No	49	24.7
Yes	149	75.3
Amount of CIT or MH Training		
One Day	9	6.0
One Week	68	45.6
Annual Training	72	48.4
Department Mental Health Unit		
Yes	127	64.1
No	71	35.9
Member of Mental Health Unit		
Yes	11	8.7
No	116	91.3
Average number of mental health crisis calls/week		
0	41	20.7
1–3	43	21.7
4–5	42	21.2
6–9	22	11.1
10–15	25	12.6
More than 15	25	12.6
	*M (SD)*	Range
Time as an officer (Years)	16.8 (9.1)	0.5–42

### Procedures

Recruited participants were directed to Qualtrics to complete an anonymous online survey. Participants identifying information was only collected for compensation purposes and not linked to their survey responses. Participants were told that the researchers were interested in understanding their perceptions of people with SMI and how they make decisions related to encounters with people with SMI. Due to the transparency of the present study’s measures and the anonymity provided by an online platform, we did not disguise the purpose of the study from participants. Soomro and Yanos [51] found that police officers openly expressed their attitudes about people with SMI and therefore social desirability effects did not appear to be a methodological concern. Participants completed a battery of survey questionnaires, IATs, demographics, and read a vignette and responded to decision making questions. The order of all measures and tasks were randomized to control for ordering effects. Attention and manipulation checks were embedded amongst the participant tasks [52]. After the participants completed all components of the study, they were debriefed and provided with the first author’s contact information should they have any questions. All study procedures were approved by an Institutional Review Board.

## Measures and materials

### Implicit bias

#### Implicit Association Tests (IATs)

Participants completed two implicit association tests (IATs) that assessed biases commonly held about individuals with mental illness– dangerousness and badness [53–56]. Both IATs utilized the same target categories: Mental Illness and Physical Illness. The mental illness items were: schizophrenia, bipolar disorder, depression, and obsessive compulsive disorder. The physical illness items were: diabetes, appendicitis, cerebral palsy, and multiple sclerosis.

The two trait categories for the badness IAT were bad and good. The bad items were: horrible, nasty, terrible, and awful. The good items were: excellent, joyful, wonderful, and great [50]. The trait categories for the dangerousness IAT were dangerous and harmless. The dangerous items were: dangerous, unsafe, violent, and aggressive. The harmless items were: harmless, safe, peaceful, and gentle [53]. We administered the IATs through a survey software (Qualtrics) using Carpenter et al.’s [57] methodology. Higher scores indicate higher levels of bias toward people with SMI. We used the scoring website provided by IATgen, the team that developed survey-software IATs, to calculate D scores.

#### The Mental Illness Microaggressions Scale-Perpetrator version (MIMS-P)

The MIMS-P (17-items, Likert scale ranging from 1 = strongly disagree to 4 = strongly agree) measured subtle stigma through endorsement of micro-aggressive behaviors towards people with SMI [58]. We calculated a total score and three subscale scores: assumption of inability, patronization, and fear of mental illness. We conceptualized the MIMS-P as an implicit measure, however the self-report aspect of the measure which requires participants to share information about their behaviors means that it may function as an explicit measure. Research has found adequate reliability and validity of this measure among college students and the general public [58].

### Explicit bias

#### Social Distance Scale (SDS)

The SDS (7-items; Likert scale ranging from 0 = definitely willing to 3 = definitely unwilling) assessed the level of willingness to associate with a person with SMI [53]. Although the original scale was given in conjunction with a vignette describing a person with SMI, we did not use a vignette to anchor participants but instead phrased the questions pertaining to people with SMI in general. This technique has been utilized in other studies and allows participants to define what mental illness means to them [59–62]. The SDS demonstrated high internal consistency in the present study (alpha = 0.87).

#### Dangerousness scale

The Dangerousness Scale (8-items; 7-point Likert scale ranging from 1 = strongly agree to 7 = strongly disagree) assesses opinions about “mental patients’” propensity for violence. For the purposes of the present study, we modified original items to reflect modern language (e.g., people with severe mental illness instead of mental patients) and more relevant current issues and examples of behavior. The new scale demonstrated strong internal consistency (alpha = 0.81). Prior studies also reported acceptable reliability [63, 64]. Construct and concurrent validity have not yet been reported.

#### Attitudes toward Mental Illness Scale (AMIS)

The AMIS (7-items; Likert scale ranging from 1 = strongly agree to 5 = strongly disagree) examines explicit attitudes toward mental illness [51]. Two subscales were developed through factor analysis: recovery and outcomes and negative stereotypes. Prior studies found acceptable reliability and validity among a national sample and police officers [51, 62]. In the present study, the AMIS total scale demonstrated a moderate level of internal consistency (alpha = 0.67), the negative stereotypes subscale demonstrated higher internal consistency (alpha = 0.71), and the recovery and outcomes subscale demonstrated the lowest level of internal consistency (alpha = 0.62).

#### Mental Health Attitude Survey for Police (MHASP)

The MHASP assesses attitudes of people with SMI via items that uniquely apply to police officers’ line of duty [65]. The MHASP consists of 33-items on a Likert scale (1 = strongly agree to 6 = strongly disagree). A four-factor model was developed with Cronbach’s alphas ranging from 0.75 to 0.89, with a Cronbach’s adjusted alpha for the total scale at 0.87. The four subscales include: positive attitude toward emotionally disturbed persons (EDPs), negative attitude toward community responsibility for EDPs, officers feel inadequately prepared to deal with EDPs, and positive attitude toward EDPs living in the community. The MHASP was validated among police officers and reliability assessments demonstrated strong internal consistency [65].

### Police department climate

#### Procedurally Just Organizational Climate (PJOC)

This is a 40-item attitudinal measure of procedural justice within a police organization [47]. The measure uses a variety of anchors for the Likert scales including 1 = not at all to 5 = a great deal and 1 = never to 5 = always. The measure includes three scales assessing different domains of a procedurally just climate: supervisor (16 items), officers (12 items), and departmental policy (12 items). Trinkner and colleagues [47] found acceptable internal consistency. In this sample, all three scales had high internal consistency (alpha ≥ 0.90).

### Familiarity with mental illness

#### Level of Contact Report (LCR)

To assess familiarity with mental illness, participants completed the LCR, a continuous measure of contact with people with SMI. The measure is a 12-item list of situations varying by degree of intimacy/closeness an individual may have had with a person with SMI. Participants were asked to check a box for all items that they have experienced, such as “I have a relative who has a severe mental illness.” These items were pre-ranked by field experts from least to most experience with mental illness. The score ranged from 1 to 12 based on endorsed items [66]. The higher the ranking the more familiarity with mental illness one has.

### Mental health crisis vignette and outcome items

#### Mental health crisis vignette

A written vignette was developed using prior vignette designs as guidance [4, 67–69]. The vignette describes an officer who encounters an individual experiencing psychiatric symptoms in an ambiguous situation that allows for officer discretion in how they respond to the incident to ensure variability in the use of force and resolution decisions. We piloted the original vignette with 18 police officers to assess the amount of discretion allowed, ambiguity of the scenario, whether they agree the person has a psychiatric/mental illness, and how realistic the encounter is in the vignette. Based on officer feedback, we made a few modifications to the wording of the use of force and resolution questions. See Appendix A for final vignette.

To enhance the ecological validity of the outcome measures and approximate real-time decision-making, officers had 15 s to respond to each question.^2^ To ensure time constraints did not negatively impact data quality, officers again responded to the Likert scale items with no time limit.

#### Use of force

Police officers were asked, *“Given the information you have at this time”* what level of response is ideal and what level of response is most likely. Following Morabito et al. [70] guidelines, officers were offered five options: “1) My mere presence, 2) Verbal warnings, commands, and/or persuasion, 3) Physical control of the suspect, such as holding, open hand strike, and/or knee strike, 4) The use of a weapon other than my firearm, such as a taser, baton, and/or chemical weapon, and 5) The use of a deadly weapon, such as my firearm.” This 5-point scale is accepted as practice in the literature and corresponds to the standard operating procedures of several police departments [e.g., 70, 71, 72, 73]. Additionally, this scale aligns with the National Institute of Justice’s use of force continuum [74].

#### Resolutions

Three items were used to assess police officers’ resolutions in response to the vignette. Police officers were asked, “*Given the information you have at this time*,* how likely would you be to arrest the civilian?; how likely would you be to take the person to a mental health facility or hospital for mental health reasons?* (referred to as mental health transport in the text); and *how likely would you be to take no formal action with the civilian described in this encounter?* (referred to as no action in the text).” Officers responded to these items on 5-point Likert (from very unlikely to very likely) scales. Items were treated as continuous variables.

Study measures and materials can be found at: https://osf.io/p3tvd/files/osfstorage/67cb59dd6aa7596e6dfd8446. 

## Results

As noted earlier, police officers responded to the same timed (15 s) and then untimed outcome items. Paired samples t-tests between timed and untimed variables were non-significant, except for between the timed and untimed mental health transport variables. The untimed mental health transport’s mean was significantly higher than the timed mental health transport (*p* =.021), when officers had more time to respond, they were less likely to transport the individual to mental health services.

Table 3 shows the frequencies for the officer decision-making variables. Officers were most likely to endorse being most likely to use non-physical levels of force in the described incident. Most officers indicated being very unlikely to arrest the individual. For mental health transport, officers were most likely to endorse neither likely or unlikely. Finally, there was a fairly even distribution of unlikely or neither likely nor unlikely on not taking formal action, followed by likely.

**Table 3:** Frequencies of (Untimed) Outcome Variables

	My mere presence (1)	Verbalwarnings	Physicalcontrol ofsuspect	Use of aweapon otherthan my firearm	Use of adeadlyweapon (5)
	*N* (%)				
Likely response	43 (21.7)	153 (77.3)	2 (1.0)	0	0
Ideal response	70 (35.4)	128 (64.6)	0	0	0

	Very Likely (1)	Likely	Neither Likely nor Unlikely	Unlikely	Very Unlikely (5)
	*N* (%)				
Arrest	3 (1.5)	2 (1.0)	25 (12.6)	60 (30.4)	108 (54.5)
Mental health transport	14 (7.1)	42 (21.2)	81 (40.9)	40 (20.2)	21 (10.6)
No formal action	21 (10.6)	47 (23.7)	56 (28.3)	56 (28.3)	18 (9.1)

Given the lack of variability of participant responses to the likely level of response, ideal level of response, and arrest questions, we did not conduct multivariate analyses for these variables. We conducted four hierarchical multivariate regressions for the mental health transport (timed and untimed) and no action variables (timed and untimed). The two models for mental health transport were similar, and we only present the results for the untimed regression.

### Multivariate analyses

For the regression analyses, we ran linear hierarchical regressions to examine the added impact of implicit biases (Step 3 in regressions: MIMS-P Total Scale; MIMS-P Fear of Mental Illness Subscale; Badness IAT; Dangerousness IAT) beyond explicit biases (Step 2 regressions: Social Distance Scale; Dangerousness Scale; AMIS Total Scale; MHASP Total Scale; MHASP Inadequately Prepared to Deal with EDPs Subscale) while controlling for demographics (Step 1 in regressions: age; gender; race; education; income; familiarity with mental illness; department size; department geographic region; CIT/mental health training; mental health unit; average weekly number of EDP calls; time as an officer). We then added procedurally just organizational climate subscales (Step 4 in regressions: Supervisors Subscale; Officers Subscale; Departmental Policies Subscale) and interactions between procedurally just organizational climate and implicit bias variables (Step 5 in regressions, which varied by model based on main effect significance). All bias variables included in the model had correlations below 0.70.^3^ We tested and confirmed that all regression assumptions, including linearity, independence of errors, homoscedasticity, normality of residuals, and absence of multicollinearity, were met.

Due to limited sample size we did not include interaction terms between all implicit bias variables and all department climate variables. The interaction terms that were included were based on significance levels and largest effect sizes of implicit bias and department climate variables. Interaction terms were created even when there were no significant main effects because of the possibility that moderation can occur when the effect of one predictor on the outcome variables is dependent on the level of another predictor [75]. Table 4 displays the linear regression results for the mental health transport and no action (timed and untimed) dependent variables.

**Table 4:** Hierarchical linear regression results of untimed mental health transport (1 = very unlikely to 5 = very likely), untimed no action (1 = very unlikely to 5 = very likely), and timed no action (1 = very unlikely to 5 = very likely)

Variable	Untimed mental health transport		Untimed no action		Timed no action	
	Exp(B) 95% CI [LL, UL]					
Age	0.00	[−0.047, 0.041]	0.03	[−0.025, 0.079]	0.02	[−0.03, 0.07]
Gender (Ref.: Male)	0.12	[−0.266, 0.513]	0.27	[−0.171, 0.719]	0.02	[−0.41, 0.44]
Race (Ref.: White)	0.00	[−0.539, 0.53]	0.01	[−0.616, 0.64]	0.18	[−0.41, 0.77]
Education (Ref.: Some College or less)						
Bachelor’s degree	0.03	[−0.365, 0.43]	−0.37	[−0.826, 0.091]	−0.46*	[−0.89, −0.02]
Post-graduate	0.18	[−0.32, 0.681]	−0.04	[−0.614, 0.542]	−0.15	[−0.70, 0.40]
Income (Ref.: Less than $75,000)						
$75,000 to $99,999	0.17	[−0.529, 0.877]	0.22	[−0.591, 1.029]	0.28	[−0.52, 1.07]
More than $100,000	−0.10	[−0.733, 0.538]	0.42	[−0.313, 1.155]	0.10	[−0.62, 0.82]
Fam. with mental illness	−0.10	[−0.217, 0.013]	0.06	[−0.069, 0.196]	0.06	[−0.07, 0.18]
Department size (Ref.: Less than 100 officers						
101–500 Officers	0.33	[−0.138, 0.793]	0.24	[−0.308, 0.78]	0.19	[−0.34, 0.72]
501–1,000 Officers	0.01	[−0.592, 0.617]	0.41	[−0.297, 1.12]	0.11	[−0.57, 0.78]
More than 1,000 Officers	−0.47	[−1.129, 0.183]	0.40	[−0.36, 1.166]	0.31	[−0.42, 1.04]
Department Geographic Region (Ref.: Suburban)						
Urban	0.57**	[0.16, 0.983]	0.11	[−0.371, 0.588]	0.10	[−0.36, 0.56]
Rural	0.42	[−0.12, 0.956]	−0.01	[−0.634, 0.605]	−0.29	[−0.88, 0.30]
CIT (Ref.: Yes)	0.13	[−0.285, 0.546]	0.17	[−0.314, 0.647]	0.12	[−0.33, 0.58]
Dept. Mental Health Unit (Ref.: Yes)	0.01	[−0.405, 0.423]	0.27	[−0.214, 0.744]	0.11	[−0.35, 0.57]
Average Number of EDP Calls/Week (Ref.: 0)						
1–3	0.13	[−0.348, 0.615]	0.26	[−0.286, 0.813]	−0.20	[−0.73, 0.33]
4–5	0.07	[−0.439, 0.581]	0.61*	[0.024, 1.196]	0.24	[−0.32, 0.79]
6–9	0.18	[−0.459, 0.818]	0.01	[−0.728, 0.745]	−0.46	[−1.18, 0.25]
10–15	−0.06	[−0.627, 0.515]	−0.15	[−0.806, 0.515]	−0.14	[−0.76, 0.48]
More than 15	−0.20	[−0.799, 0.399]	0.18	[−0.518, 0.885]	0.05	[−0.62, 0.72]
Time as an officer	−0.01	[−0.052, 0.043]	−0.03	[−0.086, 0.025]	−0.02	[−0.07, 0.03]
Explicit bias						
Social Distance Scale	−0.07**	[−0.13, −0.019]	0.01	[−0.051, 0.078]	−0.04	[−0.11, 0.02]
Dangerousness Scale	0.02	[−0.007, 0.048]	0.01	[−0.026, 0.038]	0.03	[0.00, 0.06]
AMIS Total Scale	0.01	[−0.028, 0.051]	0.06*	[0.012, 0.104]	0.03	[−0.02, 0.07]
MHASP Total Scale	0.01	[−0.01, 0.02]	0.00	[−0.016, 0.019]	0.01	[−0.01, 0.02]
MHASP Inadequately Prepared to Deal with EDPs	−0.04	[−0.112, 0.023]	−0.03	[−0.107, 0.049]	0.01	[−0.07, 0.08]
Implicit bias						
MIMS-P Total Scale	0.10	[−0.066, 0.266]	0.00	[−0.035, 0.033]	−0.01	[−0.05, 0.02]
MIMS-P Fear of MI	0.01	[−0.113, 0.133]	−0.14	[−0.278, 0.006]	−0.07	[−0.21, 0.06]
Badness IAT	−3.03*	[−5.807, −0.244]	0.30	[−2.998, 3.605]	−1.66	[−4.77, 1.44]
Dangerousness IAT	0.06	[−0.28, 0.408]	1.58	[−1.47, 4.639]	5.62***	[2.75, 8.49]
Department climate						
PJOC Supervisors	−0.01	[−0.033, 0.005]	0.01	[−0.015, 0.029]	0.00	[−0.02, 0.02]
PJOC Officers	0.05	[−0.064, 0.169]	0.04*	[0.002, 0.081]	0.08***	[0.04, 0.11]
PJOC Dept. Policies	0.01	[−0.018, 0.029]	−0.02	[−0.045, 0.009]	−0.02	[−0.05, 0.01]
Interactions						
Badness IAT*PJOC Officers	0.06*^	[0, 0.115]	0.00	[−0.07, 0.067]	0.04	[−0.02, 0.11]
Dangerousness IAT*PJOC Officers	0.00	[−0.004, 0.002]	−0.04	[−0.103, 0.024]	−0.12***	[−0.18, −0.06]
Constant	−0.40	[−7.581, 6.775]	−1.48	[−6.911, 3.947]	−2.19	[−7.32, 2.95]
Naglekerke *R*^2^	0.30		0.23		0.29	
Goodness-of-fit (df_1_,df_2_) *F*	(35, 146) 1.79		(35, 146) 1.23		(35, 142) 1.63	

This table displays the results for the final step, Step 5, of the hierarchical regressions for each dependent variable. The full model results (steps 1–5) for each regression are available upon request

*AMIS Total Scale* Attitudes About Mental Illness Total Scale, *MHASP* Mental Health Attitude Survey for Police, *MIMS-P Total Scale:*Mental Illness, Microaggression Scale– Perpetrator Version, *PJOC* Procedurally Just Organizational Climate

Note. *N* = 172

*p* <.05 *, *p* <.01 **, *p* <.001 ***

marginally significant *p* =.051 *^

#### Mental health transport (untimed)

For the untimed mental health transport dependent variable, higher scores indicate officers being more likely to transport the individual (1 = very unlikely to 5 = very likely). Notably, officers in urban areas were significantly more likely to believe they would transport the person to mental health services than those in suburban areas (B = 0.57). This may be due to more mental health resources being available in cities compared to suburbs. Although we did not capture data on specific resources in the participants’ communities, these variables may serve as a proxy to mental healthcare accessibility. Implicit bias measures contributed an additional 7% of variance explained after accounting for demographics and explicit bias measures (total *R*^2^ = 0.26 in Step 3). Procedurally Just Organizational Climate (PJOC) variables explained an additional 2% of variance in the model. In Step 4, the Social Distance Scale was a significant predictor of the likelihood of mental health transport, such that higher scores were associated with a lower likelihood of transporting the person to a hospital or mental health facility (B = −0.07). The opposite trend was observed for the MIMS-P scale, such that those more likely to endorse committing microaggression were more likely to transport the person to a facility (B = 0.05).

**Fig. 1:**
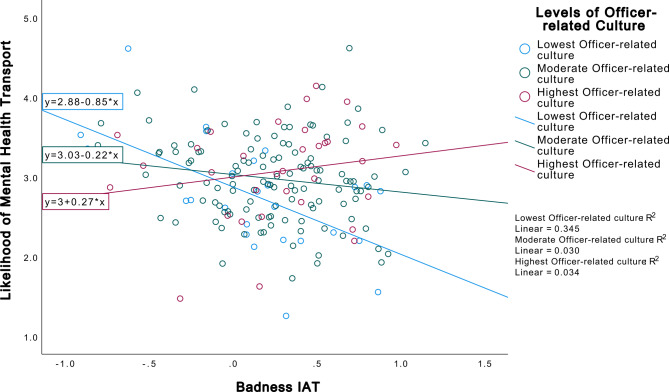
Interaction between Badness IAT and PJOC officers subscale on untimed mental health transport dependent variable

Among the department climate variables, the Officers Subscale had the largest effect size (B = 0.02). Thus, we created two interaction terms using the MIMS-P Total Scale (B = 0.05) and Badness IAT (B = −0.28) and the Officers Subscale in step 5. The interaction terms in the model increased the variance accounted for by 2% (*R*^2^ = 0.30). There was a marginally significant interaction between the Badness IAT and the Officers Subscale (B = 0.06, *p* =.051). Figure 1 demonstrates the dependency of implicit bias of people with SMI as bad on the level of perception of officer-related departmental climate on the likelihood of an officer taking the individual to a mental health facility or hospital. The interaction is in the expected direction, such that those with higher levels of bias and the lowest levels of officer-related departmental climate were the least likely to take the person to a mental health facility or hospital. In Step 5, the Social Distance Scale remained significant (B = −0.07, *p* =.009). The Badness IAT became a significant predictor, such that higher levels of implicit bias toward people with SMI as bad was associated with a 3.03 unit decrease in the dependent variable, meaning the officers were less likely to take the person to a hospital or mental health facility (B = −3.03, *p* =.033) if they implicitly associated people with SMI with badness.

#### No action (untimed)

We conducted a hierarchical multivariate linear regression for the untimed no action dependent variable (1 = very unlikely to 5 = very likely). In this model, explicit bias explained 4% more variance than demographics alone (*R*^2^ = 0.16), implicit bias only explained 3% more variance above and beyond the explicit bias variables and demographics (*R*^2^ = 0.19). PJOC variables explained an additional 3% of variance in the model (*R*^2^ = 0.22). No implicit bias variables were significant. A measure of explicit bias, the AMIS Total Scale was the only significant predictor, such that higher scores on the AMIS were associated with an increase in the dependent variable, meaning officers with greater explicit bias were more likely to take no action in the described scenario (B = 0.06, *p* =.01).

The Officers Subscale had the largest effect size in Step 4 (B = 0.03), and we chose two implicit bias measures with the largest effect size to create interaction terms with the Officer Subscale: the Badness IAT (B = 0.22) and the Dangerousness IAT (B = −0.23). With these terms included, this model accounted for an additional 1% of variance in the dependent variable (*R*^2^ = 0.23). In this model, AMIS Total Scale remained significant (B = 0.06, *p* =.014). Interestingly, the Officers Subscale became a significant predictor, such that higher officer-related departmental climate was associated with a increase in the dependent variable, meaning significantly more likely to take no action in the scenario (B = 0.04, *p* =.040). Neither interaction term was significant.

#### No action (timed)

We conducted a hierarchical linear regression for the timed no action dependent variable, where higher scores indicate being more unlikely to take no action (1 = very unlikely to 5 = very likely). In this model, implicit bias accounted for only 4% more variance above and beyond explicit bias and demographics (*R*^2^ = 0.15). PJOC variables accounted for an added 5% of variance (*R*^2^ = 0.20) in the dependent variable. The Officers Subscale was significant in Step 4, such that officers with more positive perceptions of officer-related departmental climate were more likely to take no action in the described encounter (B = 0.05, *p* =.003).

**Fig. 2:**
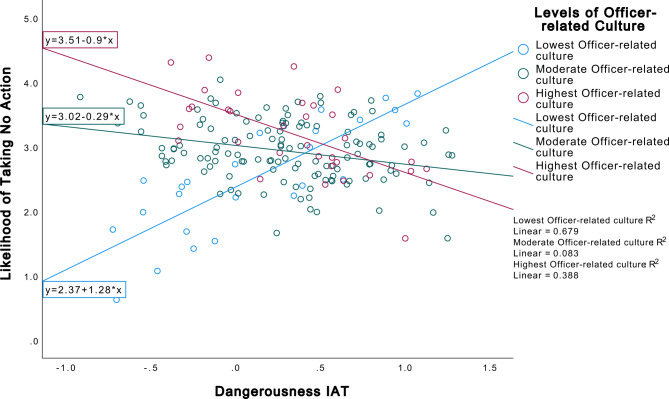
Interaction between dangerousness IAT and PJOC officers subscale on timed no action dependent variable

Because the Officers Subscale was significant in Step 4, we chose two implicit bias measures with the largest effect size to create interaction terms with the Officer Subscale: the Badness IAT (B = 0.38) and the Dangerousness IAT (B = −0.18). The inclusion of interaction terms in the model accounted for an additional 9% of variance in the dependent variable (*R*^2^ = 0.29). In this model, the Officers Subscale remained significant (B = 0.08, *p* <.001). Interestingly, the Dangerousness IAT became a significant predictor, such that higher levels of implicit bias of people with SMI as dangerous were associated with a 5.62 unit increase in the dependent variable, meaning significantly more likely to take no action in the scenario (B = 5.62, *p* <.001). There was also a significant interaction between the Dangerousness IAT and Officers Subscale (B = −0.12, *p* <.001). Figure 2 demonstrates the dependency of implicit bias of people with SMI as dangerous on the level of perception of officer-related departmental climate on the likelihood of an officer taking no action in the vignette encounter. For those with the lowest officer-related department climate, higher implicit bias of people with SMI as dangerous was associated with being more likely to not take action in the encounter.

## Discussion

The present study sought to better understand how bias toward mental illness and police organizational climate impact police officer decision-making in a mental health crisis using a vignette design. Although research in several domains has demonstrated the consequences of bias and the impact of bias on decision-making, the extant literature has yet to examine the differential role of implicit and explicit bias towards people with SMI in police officer decision-making. Research has supported the role of racial bias in harmful and disparate policing outcomes [76]. People with SMI are another vulnerable population when it comes to police interactions, and thus the present study helps to expand the literature in this area through identifying how mental illness bias may be related to police officer decision-making in mental health crises. Moreover, we consider both individual and systemic factors that may contribute to negative policing outcomes by examining the influence of departmental climate on the relationship between bias toward people with SMI and officer decision-making. Below we discuss our findings, first related to arrest and use of force, followed by transport and no action outcomes.

### Arrest and use of force

In this study, few officers indicated that physical force or arrest would be a likely outcome when responding to the vignette scenario. Indeed, only two officers endorsed any use of physical force as likely or ideal in response to the vignette. Similarly, only five (2.5%) participants indicated that it would be likely or very likely that the encounter would result in an arrest. Although use of force and arrests are relatively low frequency events overall, there is evidence that people with SMI experience an increased risk of use of force and are arrested at higher rates for lower-level offenses compared to those without mental illness [19, 24, 25, 77]. The study’s vignette-based methodology, which required officers to *simulate* their decision-making based on a limited amount of information, may explain the low prevalence of use of force and arrest in the vignette encounter. Specifically, they were asked to indicate the level of force and likelihood of different resolutions based on “the information you have at this time.” The vignette does not contain specific information indicating a crime has occurred or enough detail to determine if physical force may be warranted. Participants may have responded strictly based on the information provided rather than considering how the encounter was likely to unfold. Thus, because no information was explicitly given to justify use of force, nor information about a criminal offense, this may have influenced officers' judgements, which would likely differ from their actual behaviors. We speculate social desirability may have been an influencing factor on officers’ responses in this study. Therefore, these descriptive findings must be interpreted with caution as they do not reflect actual outcomes in mental health crises. 

### Implicit bias & officer decision-making

We hypothesized implicit bias would significantly predict officer decision-making, over and above explicit bias. Prior research has found that although the effects of implicit bias on behavior are typically small, they are often more consistent and more significant than the effects of explicit bias [34–36]. Studies specifically examining the effect of implicit bias on behavior toward people with SMI have found that implicit bias predicts over-diagnosing mental illness [38], invasive medication monitoring, and involuntary hospitalization [40] among mental health staff.

In line with prior research demonstrating stronger predictive validity of IATs than explicit measures, implicit bias accounted for more variance in officer decision-making than explicit bias. However, implicit bias measures were not consistently significant predictors of decision-making, nor were explicit bias measures. Higher levels of implicit bias of people with SMI as bad were associated with being *less* likely to transport the individual to mental health services. Similarly, higher scores on the Social Distance Scale were associated with being *less* likely to transport the individual to mental health services. Both make sense given that, if officers view a person with SMI as bad and have less desire to interact with the person, then presumably they would be less likely to see a need for care or have a desire to interact with them longer than necessary to transport them to services. In contrast, greater endorsement of microaggressions toward mental illness were associated with officers being *more* likely to take the person in crisis to a hospital or mental health facility. This finding regarding microaggressions may be similar to other studies that showed clinicians with higher implicit biases toward people with SMI were associated with more controlling and restrictive clinical interventions, such as hospitalization [40]. These findings also align with a benevolence stigma, a negative and paternalistic belief toward serious mental illness in which people believe people with SMI need to be cared for [65].

The Dangerousness IAT was a significant predictor of no action, such that higher levels of implicit bias of people with SMI as dangerous were associated with officers being significantly more likely to take no action in the encounter. Given that officers were not inclined to take action, their implicit bias of dangerousness appears to be based more on their own personal feelings/views rather than information provided in the vignette. Likewise, officers with greater bias measured by the AMIS, which assesses attitudes toward mental illness more generally, were more likely to take no action in the scenario, indicating that higher levels of bias may lead to not wanting to deal with a mental health crisis.

These results demonstrate that different domains of bias may have different behavioral implications. Biases are complex, and intertwined constructs where multiple biases may co-exist within individuals. Thus, without an understanding of this complexity, interventions that target bias reduction may be limited in their long-term impacts on behavior and decision-making. Further, policies that focus on implementing resources that make it as easy as possible for officers to connect individuals to services without an undue burden may be fruitful for improving outcomes for people with SMI. These resources may include crisis drop off centers that do not require lengthy stays for officers, mental health teams such as mobile crisis that officers can directly connect citizens to, or dispatching mental health/community safety response teams in place of law enforcement [78, 79].

### Implicit bias, police departmental climate, & officer decision-making

We hypothesized police department organizational climate would moderate the relationship between implicit bias and officer decision-making, such that officers with the poorest perceptions of departmental climate and highest levels of implicit bias would endorse more negative outcome decisions in a mental health crisis. Research on police departmental climate has demonstrated when officers perceive a positive, procedurally just climate, they are more likely to take on extra work, help fellow officers, and had fewer complaints from citizens [45, 46, 48]. Although prior research has examined the effect of police departmental climate on behavior, no studies have assessed this construct in the context of mental health crises or how it may relate to implicit bias. In the present study, interactions between implicit bias and departmental climate revealed that officers with the highest levels of implicit bias and lowest levels of officer-related departmental climate, specifically more negative perceptions of procedurally just experiences with other officers, were the least likely to transport a person to services and to take action — meaning, this combination of factors led to more inaction from officers. These findings comport with prior literature that officers with worse perceptions of departmental climate are less willing to take on additional work [46], and provide insight into the relationship between police departmental climate and bias when responding to mental health crises. It appears important that officers have positive relationships and support from their colleagues in order to best serve their communities and people in crisis.

### Limitations

Although this study contributes to the field in several ways, it has limitations which must be considered when interpreting its findings. The first limitation of this study is a small sample size, which may result in Type II error. Further, although prior research has shown police officers are willing to openly disclose their attitudes toward mental illness, the study may still be prone to social desirability effects. Although we did not explicitly state that we would assess the relationship between bias and decision-making, officers may have perceived this, which may have impacted responses. The present study also did not assess availability of resources that officers are able to connect individuals to, this would further help determine how officers decide to take action or transport someone to services.

The generalizability of the study is also limited in terms of racial/ethnic diversity, range of police departments, and departmental size. Further, because each police department is unique, it is challenging to accurately capture and reflect how officer attitudes and mental health crisis decision-making may operate across departments. Understanding organizational climate within specific departments would be more informative for policy at a local level by understanding how departmental climate is perceived in particular departments. Also, due to the online, experimental nature of the study, the ecological validity is limited. Thus, how officers reported they would respond in the described vignette, may not be an accurate approximation of their behavior in real-life mental health crises. Further, the vignette only captures one type of interaction and thus these findings may not apply to situations with different circumstances, and the vignette does not capture the full dynamic of actual encounters.

### Future directions

Attitudes toward mental illness and how they may relate to police officer decision-making in mental health crises are important areas of research that need further study. Future research should utilize multiple vignettes depicting different types of crisis situations, rather than just one. This may provide a better idea of officer decision-making across crisis incidents with varying presenting problems. Showing officers videos of encounters would help to improve ecological validity even more. Further, it would be useful to also administer non-mental health crisis vignettes to provide a comparison of how officers respond in crisis situations versus non-crisis situations. However, to best demonstrate the relationship between officer decision-making and bias toward people with SMI, future research should attempt to link officer records of how they respond to actual mental health crises in the field with officer responses to bias measures. Future research should also explore how to better measure officer decision-making in mental health crises. For example, it may be useful to provide officers with a continuous sliding scale from 0 to 100 on which they could indicate their likelihood of responding to situations in a certain way. This may provide more variability in responses and allow for a more accurate assessment of officer decision-making. Although these quantitative findings identify important relationships between officer attitudes, implicit bias, organizational climate, and decision-making, future research should incorporate qualitative methods. Interviews or focus groups with officers and individuals with lived experience can provide additional insight on nuances of these patterns, including reasons behind officer behavior, the role of police departmental climate in shaping responses, and the personal experiences of mental health crises, ultimately guiding more effective training and policy development.

## Conclusion

Amidst national law enforcement staffing shortages, conversations around what services should be invested in, and developing more appropriate responses to mental health crises, findings from this study can be used to inform how governments and departments move forward in addressing mental health crises as a public health concern. Golden and Earp argue for the need of a social ecological approach to public health interventions which incorporate multiple levels of McLeroy’s ecological model for health promotion. Largely, interventions have focused on efforts to make change at the individual level, rather than aiming to modify organizational, community, and policy-level factors [10, 80]. Importantly, literature on officer-level interventions to address bias has demonstrated limited effectiveness.

Despite promising short-term gains, existing anti-stigma and implicit-bias trainings for police officers have yielded inconsistent, fleeting effects and their lasting impact on real-world decision-making remains largely untested. Tartaro and colleagues [81] discuss how although some research has found police officers have experienced benefits from anti-stigma interventions [82], research does not indicate that these positive outcomes are enduring or if they impact officer behavior and decision-making in mental health crises. Indeed, findings from research on implicit bias interventions has been weak, inconsistent, and ephemeral [31]. The strongest evidence for the potential of implicit bias interventions for durable impacts on implicit bias are for multi-session trainings [83] and prolonged interventions [84, 85] involving interactive real-world exposures that violate expectations and pre-existing associations (i.e., mental illness and dangerousness). Group trainings, which have been widely adopted by corporations to improve equity in the workplace, and single-session interventions have shown limited effectiveness often with observed differences only immediately after the intervention [31]. Notably, at present only seven studies have evaluated the impact of implicit bias training for police officers, and only two assessed impacts on behavioral change. Although trainings appeared to show short-term or immediate improvement in police officers’ understanding of implicit bias, concerns of discrimination, and intentions to use intervention strategies, long lasting impacts were either not measured or did not persist. Regarding behavior change, James et al. [86] found a positive impact of a training that combined both a didactic and simulation-based training on how officers treated individuals, although there was not a race-based difference in improvements, and only a slight decrease in discrimination-based complaints when analyzing interactions in the field. Worden et al. [33] assessed the impact of a single-session, 8-hour NYPD racial implicit bias training on officers’ beliefs, attitudes, and enforcement disparities of stops, frisks, searches, arrests, summonses, and use of force. The training showed an immediate impact on officer knowledge, but with rapid decline. Training overall did not reduce disparities in enforcement, with only weak support for an impact on use of force racial disparities for Latino suspects. Overall, the behavioral impacts of implicit bias trainings on officers are largely under examined. Research suggests that when considering interventions to address bias and discriminatory behavior, law enforcement agencies will likely see the least benefit from didactic-based, single-session interventions, and may benefit more from strategies that integrate exposure to individuals from the target group in natural ways in the field. For example, pairing female STEM college students with female STEM senior mentors and white college students with Black roommates demonstrated improvements in implicit association tests with lasting effects [84, 85].

Our findings suggest the impact of bias on police officer decision-making in encounters with people with SMI is complex, thus, bias reduction efforts must address this complexity. In line with prior research, programs to address how communities respond to mental health crises would likely benefit from integrating peer specialists as ongoing community advisors who meet directly with officers, as co-responders and first responders to combat stigma and discriminatory behavior through association-breaking exposure with colleagues who have lived experience of mental illness and criminal legal involvement. Importantly, factors which impact mental health crises resolutions are widespread. Systemic factors and resources must be critically evaluated when attempting to address challenging societal issues.

When considering police reform, police departmental climate must also be considered as a potential barrier and facilitator of change. The 2015 Presidential Task Force on 21st century policing describes that “organizational culture eats policy for lunch” (2015, p. 11; [7, 87]). This begs the question, how can departmental climate be overcome? Police departments may consider recommendations to achieve structural change in how police engage with communities more broadly [88], and shift how mental health calls are discussed, especially with new officers in training. Further research is needed to understand the efficacy of organizational climate interventions in law enforcement agencies. While organizational-level interventions may help to make strides in how mental health crises are resolved, policy changes outside of law enforcement agencies may be more effective in materializing better outcomes for people with SMI. Therefore, rather than just focusing on *how* we respond to mental health crises, it may be more effective to consider *who* responds.

A multipronged social ecological approach is needed to create lasting population-level change and re-envision the first response landscape for mental health needs [10, 80]. Informed by Golden and Earp’s review of social ecological approaches, we present several multi-level intervention strategies while recognizing that resources are often limited. However, by removing the emphasis on police officers and departments, other systems can take on responsibility for implementing programs to improve how communities respond to crises. At the individual level, we propose integrating bias interventions grounded in interactive, real-world exposures directly into training curricula, with pre‐ and longitudinal post‐assessments to assess the long-term value of such initiatives and adapt trainings accordingly. Simultaneously, at the organizational level, departments should adopt clear policy directives to complement these trainings (i.e., ongoing peer‐coaching and leadership feedback loops, and development of crisis call protocols with regular review).

At the community and policy level, active partnerships with local mental health providers and offloading a range of call types to alternative community safety response or mobile crisis teams can help alleviate police department burden and potential negative policing outcomes while simultaneously building public health infrastructure. Since 2020, unarmed, non-police response teams, composed of mental health professionals, EMTs, and/or peer specialists, have begun to proliferate across the US. These alternative community safety response programs to respond to mental health crises may help to better connect those suffering with mental illness to services in the community. Indeed, both officers and persons in crisis may benefit most from utilization of other resources and community-based solutions to address mental health needs in the community. For jurisdictions that continue to rely on police officers to address mental health crises, implicit bias can be conceptualized as a public health issue. Thus, public health solutions, such as engaging in demographic disparity finding through data usage to assess differences in policing outcomes based on group membership, can work to make such patterns visible at both the individual and organizational levels [31, 33]. Departments must also consider investing in developing relationships with mental health organizations, and developing the infrastructure needed to facilitate law enforcement’s role in connecting individuals to mental health services. It may be beneficial for local governments and communities to thoroughly examine availability and accessibility of mental health resources in their communities to identify gaps in services and develop a more robust mental health care system. Jurisdictions can take a multi-dimensional approach drawing from preventive, governmental, and reparative based solutions [31]. Community involvement and assessments of these interventions can help ensure these programs are having their intended effects at the community level. Ongoing research and evaluation efforts will help ensure interventions not only reduce stigma, but translate into safer, more equitable interactions in practice. Without renewed approaches, all first responses will be limited by the services available in their communities which are ultimately responsible for longer term care and treatment.

## Supplementary Information

Supplementary Material 1.

Supplementary Material 2.

## Data Availability

Availability of data and materials: Data collected in this study is not yet publicly available but is available upon request from first author. First author will make data publicly available on Open Science Framework following publication of study’s primary manuscripts. Study measures are available at https://osf.io/p3tvd/files/osfstorage/67cb59dd6aa7596e6dfd8446.

## Abbreviations

SMI: serious mental illness
IAT: Implicit Association Test
ICC: implicit-criterion-correlation
ACT: Assertive Community Treatment
CIT: Crisis Intervention Team
EDP: emotionally disturbed person
MIMS-P: Mental Illness Microaggressions Scale-Perpetrator Version
SDS: Social Distance Scale
AMIS: Attitudes toward Mental Illness Scale
MHASP: Mental Health Attitude Survey for Police
PJOC: Procedurally Just Organizational Climate
LCR: Level of Contact Report

## Footnotes

1 Two participants identified as other and were excluded from the multivariate analyses.

2 Fourteen participants answered the questions in 10 s or less and based on their feedback we extended the time to 15 s.

3 The bias measures not included in these analyses were: AMIS Negative Stereotypes, AMIS Recovery & Outcomes, MHASP Negative Attitude toward Community Responsibility, and MHASP Positive Attitude toward Community Living, MHASP Positive Attitude toward EDPs, MIMS-P Assumption of Inability, and MIMS-P Patronization.
