# Supplementary material for: Responding to crisis: the intersection of police officer attitudes, organizational climate, and mental health crises
Source: BMC Public Health. 2025 Oct 7;25:3371. doi: 10.1186/s12889-025-23832-8 (PMC12502404; doi:10.1186/s12889-025-23832-8)
Supplement: Supplementary file 1 — Supplementary Material 1. [file 12889_2025_23832_MOESM1_ESM.docx]

Study Measures & Materials

### Demographic and Background Questions

General Demographics:

1. What gender do you identify as?
   1. Male
   2. Female
   3. Trans-male
   4. Trans-female
   5. Other: ___________
2. What is your age? _______
   1. Please select your age category:
      1. 18 – 29
      2. 30 – 39
      3. 40 – 49
      4. 50 – 59
      5. 60+
3. Do you identify as Hispanic or Latino/a/x?
   1. Yes
   2. No
4. What race do you identify as?
   1. White
   2. Black or African American
   3. Asian/Pacific Islander
   4. Native American
   5. Biracial
   6. Other: _________________________________
5. What is your educational background?
   1. Less than high school
   2. High school or GED
   3. Some college/Associate’s degree
   4. Technical/Vocational/Professional schooling
   5. Bachelor’s degree
   6. Post-graduate degree
      1. Degree and subject area: ______________________
6. What is your yearly household income?
   1. Less than $25,000
   2. $25,000 – $49,999
   3. $50,000 - $74,999
   4. $75,000 - $99,999
   5. More than $100,000
7. What state do you live in?
   1. Drop down menu option
8. How would you describe the geographic region in which you live?
   1. Urban
   2. Suburban
   3. Rural

Police Officer Demographics:

1. How long have you been a police officer?
   1. ________years _______months
2. How large is your police department?
   1. Less than 100 officers
   2. 101 – 500 officers
   3. 501 – 1000 officers
   4. 1001 – 1500 officers
   5. 1501 – 2000 officers
   6. 2000+
3. How would you best describe your police department?
   1. Urban
   2. Suburban
   3. Rural
4. Have you undergone Crisis Intervention Team (CIT) training?
   1. Yes
   2. No
5. IF YES, how much training did you receive?
   1. One day
   2. One week
   3. Annual training
      1. Number of years receiving annual training: ___________
   4. CIT Certification
6. Does your department have a specific Crisis Intervention Team/Unit?
   1. Yes
   2. No
7. IF YES, are you a member of the team/unit?
   1. Yes
   2. No
8. On average, approximately how many crisis/mental/or EDP calls do you believe you respond to each week?
   1. Zero
   2. 1 – 3
   3. 4 – 5
   4. 6 – 9
   5. 10 – 15
   6. More than 15

### Familiarity with Mental Illness

Holmes EP, Corrigan PW, Williams P, Canar J, Kubiak MA. Changing attitudes about schizophrenia. Schizophrenia bulletin. 1999 Jan 1;25(3):447-56.

Instructions to participants: Please read each of the following statements carefully. After you have read all the statements below, place a check by the statements that best depict your exposure to persons with a severe mental illness.

| ______ | I have watched a movie or television show in which a character depicted a person with mental illness. |
| --- | --- |
| ______ | My job involves providing services/treatment for persons with a severe mental illness. |
| ______ | I have observed, in passing, a person I believe may have had a severe mental illness. |
| ______ | I have observed persons with a severe mental illness on a frequent basis. |
| ______ | I have a severe mental illness. |
| ______ | I have worked with a person who had a severe mental illness at my place of employment. |
| ______ | I have never observed a person that I was aware had a severe mental illness. |
| ______ | My job includes providing services to persons with a severe mental illness. |
| ______ | A friend of the family has a severe mental illness. |
| ______ | I have a relative who has a severe mental illness. |
| ______ | I have watched a documentary on the television about severe mental illness. |
| ______ | I live with a person who has a severe mental illness. |

### The Mental Illness Microaggressions Scale – Perpetrator Version

Gonzales L, Davidoff KC, DeLuca JS, Yanos PT. The mental illness microaggressions scale-perpetrator version (MIMS-P): Reliability and validity. Psychiatry Research. 2015 Sep 30;229(1-2):120-5.

1-4 Likert Scale: 1 = strongly disagree, 2 = disagree, 3 = agree, 4 = strongly agree

1. If someone I’m close to told me that they had a mental illness diagnosis, I would expect them to have trouble understanding some things.
2. If someone I’m close to told me that they had a mental illness diagnosis, I would try to talk more slowly so that they wouldn’t get confused.
3. If someone I’m close to told me that they had a mental illness diagnosis, I would avoid asking them for favors because it would be hard for them to help someone else.
4. If someone I’m close to told me that they had a mental illness diagnosis, I would be careful in case they “snap.”
5. If someone I’m close to told me that they had a mental illness diagnosis, I would tell them they should go to the hospital, even if they told me they were fine.
6. If someone I’m close to who I thought was normal told me that they had a mental illness diagnosis, I’d tell them they were probably exaggerating.
7. If someone I’m close to told me that they had a mental illness diagnosis, I would be confused if I saw them behaving normally.
8. If someone I’m close to told me that they had a mental illness diagnosis, I would keep my distance from them.
9. If someone I’m close to told me that they had a mental illness diagnosis, I would encourage them to keep it a secret.
10. If someone I’m close to told me that they had a mental illness diagnosis, I would give them advice on how to deal with their illness.
11. If someone I’m close to told me that they had a mental illness diagnosis, I would tell them when I thought their behavior was caused by symptoms.
12. If someone I’m close to told me that they had a mental illness diagnosis, I would frequently remind them that they need to take their medication.
13. If someone I’m close to told me that they had a mental illness diagnosis, I would give them advice on how to remain stable.
14. If someone I’m close to told me that they had a mental illness diagnosis, I would help by telling them when they’re showing signs of their illness.
15. If someone I’m close to told me that they had a mental illness diagnosis, I would look out for specific symptoms and behaviors.
16. If I saw a person who I thought had a mental illness in public, I would keep my distance from them.
17. If I saw a person who I thought had a mental illness in public, I would be careful in case they “snap.”

### Explicit Bias Measures

#### Social Distance Scale.

Penn DL, Guynan K, Daily T, Spaulding WD, Garbin CP, Sullivan M. Dispelling the stigma of schizophrenia: what sort of information is best?. Schizophrenia Bulletin. 1994 Jan 1;20(3):567-78.

Instructions to participants: Please rate the following statements on the following scale: 0 = definitely willing; 1 = probably willing; 2 = probably unwilling; 3 = definitely unwilling.

1. How would you feel about renting a room in your home to someone with a mental illness?
2. How about as a worker on the same job as someone with a mental illness?
3. How would you feel having someone with a mental illness as a neighbor?
4. How about as the caretaker of your children for a couple of hours?
5. How about having your children marry someone with a mental illness?
6. How would you feel about introducing someone with a mental illness to a young woman you are friendly with?
7. How would you feel about recommending someone with a mental illness for a job working for a friend of yours?

#### Dangerousness Scale.

Penn DL, Kommana S, Mansfield M, Link BG. Dispelling the stigma of schizophrenia: II. The impact of information on dangerousness. Schizophrenia Bulletin. 1999 Jan 1;25(3):437-46.

Note: Original scale was revised to use updated language and examples. See revisions in red. Revised scale demonstrated strong internal consistency in a nationally representative Prolific sample and a sample of police officers in the US.

Participants responded to each item on a 7-point Likert scale ranging from 1 (strongly agree) to 7 (strongly disagree).

1. If a group of *former mental patients* lived nearby, I would not allow my children to *go to the movie theatre alone.

Revision: people with severe mental illness; to go out alone.

1. If a *former mental patient* applied for a teaching position *at a grade school* and was qualified for the job, I would recommend hiring him/her. **(Reverse scored)**

Revision: person with a severe mental illness; at an elementary school

1. One important thing about *mental patients* is that you cannot tell what they will do from one minute to the next.

Revision: people with severe mental illness

1. If I know a person has *been a mental patient*, I will be less likely to trust him.

Revision: a severe mental illness

1. The main purpose of mental hospitals should be to protect the public from mentally ill people.
2. If a *former mental patient* lived nearby, I would not hesitate to allow young children *under my care on the sidewalk. **(Reverse scored)**

Revision: person with severe mental illness; under my care to play outside in the neighborhood.

1. Although some *mental patients* may seem all right, it is dangerous to forget for a moment that they are mentally ill.

Revision: people with severe mental illness

1. There should be a law forbidding a *former mental patient* the right to obtain *a hunting license.

Revision: person with severe mental illness; a gun license.

#### Attitudes toward Mental Illness and its Treatment Scale (AMIS)

Kobau R, DiIorio C, Chapman D, Delvecchio P, SAMHSA/CDC Mental Illness Stigma Panel Members. Attitudes about mental illness and its treatment: Validation of a generic scale for public health surveillance of mental illness associated stigma. Community Mental Health Journal. 2010 Apr;46:164-76.

Instructions to participants: “In this section, there are a number of statements with which you may or may not agree. For each statement listed, please indicate whether you personally agree or disagree with it. If you don’t understand a statement or it is not applicable to you, please leave that row blank.”

All respondents will be asked to indicate level of agreement on a 5-point Likert scale, where 1 = strongly disagree, and 5 = strongly agree.

1. I believe a person with mental illness is a danger to others
2. I believe a person with mental illness is unpredictable
3. I believe a person with mental illness is hard to talk with
4. I believe a person with mental illness has only himself/herself to blame for his/her condition
5. I believe a person with mental illness would improve if given treatment and support
6. I believe a person with mental illness feels the way we all do at times
7. I believe a person with mental illness could pull himself/herself together if he/she wanted
8. I believe a person with mental illness can eventually recover
9. I believe a person with mental illness can be as successful at work as others
10. Treatment can help people with mental illness lead normal lives
11. People are generally caring and sympathetic to people with mental illness

#### Mental Health Attitude Survey for Police

Clayfield JC, Fletcher KE, Grudzinskas AJ. Development and validation of the mental health attitude survey for police. Community Mental Health Journal. 2011 Dec;47:742-51.

*The statements below represent attitudes or opinions you may have about mental illness and about dealing with emotionally disturbed persons or EDPs (a term widely used in policing to describe calls involving persons with mental illness). For each statement, please select the one response that best reflects your opinion. There are no right or wrong answers.*

|  | **Strongly Agree** | **Moderately Agree** | **Slightly Agree** | **Slightly Disagree** | **Moderately Disagree** |   **Strongly Disagree** |
| --- | --- | --- | --- | --- | --- | --- |
| **1. Emotionally disturbed persons take up more than their fair share of police time.** | **1** | **2** | **3** | **4** | **5** | **6** |
| **2. As soon as a person shows signs of mental disturbance, he/she should be hospitalized.** | **1** | **2** | **3** | **4** | **5** | **6** |
| **3. Emotionally disturbed persons need the same kind of control and discipline as a young child.** | **1** | **2** | **3** | **4** | **5** | **6** |
| **4. Residents should accept the location of mental health facilities in their neighborhood to serve the needs of the local community.** | **1** | **2** | **3** | **4** | **5** | **6** |
| **5. It is frightening to think of emotionally disturbed persons living in residential neighborhoods.** | **1** | **2** | **3** | **4** | **5** | **6** |
| **6. More tax money should be spent on the care and treatment of emotionally disturbed persons.** | **1** | **2** | **3** | **4** | **5** | **6** |
| **7. We have a responsibility to provide the best possible care for emotionally disturbed persons.** | **1** | **2** | **3** | **4** | **5** | **6** |
| **8. Nowadays, police officers need to have specialized training in dealing with emotionally disturbed persons.** | **1** | **2** | **3** | **4** | **5** | **6** |
| **9. It is best to avoid anyone who is emotionally disturbed.** | **1** | **2** | **3** | **4** | **5** | **6** |
| **10. One of the main causes of mental illness is a lack of self-discipline and will power.** | **1** | **2** | **3** | **4** | **5** | **6** |
| **11. It would be foolish to marry a person who has suffered from a mental illness, even though s/he seems fully recovered.** | **1** | **2** | **3** | **4** | **5** | **6** |
| **12. I would not want to live next door to someone who has been emotionally disturbed.** | **1** | **2** | **3** | **4** | **5** | **6** |
| **13. Residents have nothing to fear from people coming into their neighborhood to obtain mental health services.** | **1** | **2** | **3** | **4** | **5** | **6** |
| **14. Mental health facilities should be kept out of residential neighborhoods.** | **1** | **2** | **3** | **4** | **5** | **6** |
| **15. Emotionally disturbed persons should be isolated from the rest of the community.** | **1** | **2** | **3** | **4** | **5** | **6** |
| **16. Locating mental health facilities in a residential area downgrades the neighborhood.** | **1** | **2** | **3** | **4** | **5** | **6** |
| **17. Dealing with emotionally disturbed persons should be an integral part of community policing.** | **1** | **2** | **3** | **4** | **5** | **6** |
| **18. I feel that I am adequately trained to handle situations/calls involving emotionally disturbed persons.** | **1** | **2** | **3** | **4** | **5** | **6** |
| **19. Emotionally disturbed persons should not be given any responsibility.** | **1** | **2** | **3** | **4** | **5** | **6** |
| **20. There is something about emotionally disturbed persons that makes it easy to tell them apart from normal people.** | **1** | **2** | **3** | **4** | **5** | **6** |
| **21. Responding to calls involving emotionally disturbed persons is not really part of a police officers’ role.**   | **1** | **2** | **3** | **4** | **5** | **6** |
| **22. We need to adopt a far more tolerant attitude toward emotionally disturbed persons in our society.** | **1** | **2** | **3** | **4** | **5** | **6** |
| **23. Emotionally disturbed persons are a disadvantaged group who deserve special consideration from the police.** | **1** | **2** | **3** | **4** | **5** | **6** |
| **24. Locating mental health services in residential neighborhoods does not endanger local residents.** | **1** | **2** | **3** | **4** | **5** | **6** |
| **25. I feel more comfortable responding to EDP calls involving females in crisis.** | **1** | **2** | **3** | **4** | **5** | **6** |
| **26. Local residents have good reason to resist the location of mental health services in their neighborhood.** | **1** | **2** | **3** | **4** | **5** | **6** |
| **27. Increased spending on mental health services is a waste of tax dollars.** | **1** | **2** | **3** | **4** | **5** | **6** |
| **28. I know when to implement an application for emergency commitment.** | **1** | **2** | **3** | **4** | **5** | **6** |
| **29. Having emotionally disturbed persons living within residential neighborhoods might be good therapy but the risks to residents are too great.** | **1** | **2** | **3** | **4** | **5** | **6** |
| **30. There is pressure from my department to solve the problems associated with emotionally disturbed persons on an informal basis.** | **1** | **2** | **3** | **4** | **5** | **6** |
| **31. I feel confident in my ability to handle situations involving emotionally disturbed persons.** | **1** | **2** | **3** | **4** | **5** | **6** |
| **32. If mental health services were adequate, the police would not have to deal with emotionally disturbed persons.** | **1** | **2** | **3** | **4** | **5** | **6** |
| **33. There is pressure from emergency room personnel to solve the problems associated with emotionally disturbed persons on an informal basis.** | **1** | **2** | **3** | **4** | **5** | **6** |

### Procedurally Justice Organizational Climate.

Trinkner R, Tyler TR, Goff PA. Justice from within: The relations between a procedurally just organizational climate and police organizational efficiency, endorsement of democratic policing, and officer well-being. Psychology, Public Policy, and Law. 2016 May;22(2):158.

All items were responded to on a 5-point Likert scale.

Supervisor:

1. How impartial are your supervisor(s) when making decisions that affect you?
2. How much do your supervisor(s)’ prejudices influence him or her when making decisions that affect you? **(Reverse coded)**
3. How often do your supervisor(s) treat you the same way they treat everyone else when making decisions?
4. How often are the decisions that your supervisor(s) make that affect you based on facts, not personal biases?
5. How respectful are your supervisor(s) of you as a person?
6. How rough are your supervisor(s) with you when trying to get you to do what they want? **(Reverse coded)**
7. How often do your supervisor(s) treat you with dignity and respect?
8. How often are your supervisor(s) disrespectful toward you? **(Reverse coded)**
9. How often do your supervisor(s) give you explanations for the decisions they make that affect you?
10. How often do your supervisor(s) tell you the reasons for their personnel decisions?
11. How often do you know the reasons why your supervisor(s) make decisions that affect you?
12. How often do your supervisor(s) take the time to explain when they make decisions that affect you?
13. How often do your supervisor(s) show an interest in what you have to say?
14. How often do your supervisor(s) ask your opinion before making decisions that affect you?
15. How often do your supervisor(s) take the time to listen when you express your views?
16. How often do you have open dialogue with your supervisors?

Officers:

1. How even-handed are officers in this department in terms of how they treat you?
2. How much do the prejudices of officers in this department influence them when they interact with you? **(Reverse coded)**
3. How often do officers in this department treat you the same way they treat other officers?
4. How often do officers in this department let their personal biases influence the way they treat you? **(Reverse coded)**
5. How much do officers in this department respect you as a person?
6. How much consideration do officers in this department show you?
7. How often do officers in this department treat you kindly?
8. How often do officers in this department treat you with dignity and respect?
9. How often do officers in this department care about what you have to say?
10. How often do officers in this department talk freely with you?
11. How often do officers in this department ask for your opinion on issues?
12. How often do officers in this department show an interest in what you have to say?

Department Policy:

1. How fairly are the regulations defining officer misconduct applied in this department?
2. How fairly would you be treated in a formal disciplinary investigation?
3. Overall, how unfair is the disciplinary process in this department?
4. How concerned are you that you will be punished for making an honest mistake? **(Reverse coded)**
5. How fairly are good assignments assigned in this department?
6. How fairly are bad assignments assigned in this department?
7. How fairly are job assignments given out in this department?
8. How fairly are special assignments handed out among the other officers and you?
9. How concerned are you that the way assignments are handed out is unfair? **(Reverse coded)**
10. How fair are the officer promotion procedures in this department?
11. How fairly are promotions given in this department?
12. Overall, how unfair is officer promotion in this department? **(Reverse coded)**
