# Supplementary material for: Responding to crisis: the intersection of police officer attitudes, organizational climate, and mental health crises
Source: BMC Public Health. 2025 Oct 7;25:3371. doi: 10.1186/s12889-025-23832-8 (PMC12502404; doi:10.1186/s12889-025-23832-8)
Supplement: Supplementary file 2 — Supplementary Material 2. [file 12889_2025_23832_MOESM2_ESM.docx]

Appendix A. Vignette of police encounter with person experiencing a mental health crisis.

***7:46 pm*** *Police dispatch receives a call regarding a man acting erratically and in an unusual nature. Dispatch indicates this has been going on for about 15 minutes.*

***7:52 pm*** *You arrive at the scene to an unidentified man walking down the middle of a shopping center parking lot. The man appears to be talking to himself, is constantly changing the direction he is walking, and seems agitated. You notice he is holding something in his hand, but it is unclear what it is. You attempt to get his attention, but the man does not respond. The area is not crowded, but those who have passed by are clearly uncomfortable with the man’s demeanor and you’ve received complaints from the store. The man now starts walking toward you.*
